# Supplementary material for: Expression of divergent methyl/alkyl coenzyme M reductases from uncultured archaea
Source: Commun Biol. 2022 Oct 20;5:1113. doi: 10.1038/s42003-022-04057-6 (PMC9584954; doi:10.1038/s42003-022-04057-6)
Supplement: Supplementary file 2 — Supplementary Information [file 42003_2022_4057_MOESM2_ESM.docx]

**Supplementary Information:**

**Expression of divergent methyl/alkyl coenzyme M reductases from uncultured archaea**

Nana Shao^1^, Yu Fan^2^, Chau-Wen Chou^3^, Shadi Yavari^4^, Robert V. Williams^3^, I. Jonathan Amster^3^, Stuart M. Brown^5^, Ian J. Drake^6^, Evert C. Duin^4^, William B. Whitman^1*^ and Yuchen Liu^5*^

^1^Department of Microbiology, University of Georgia, Athens, Georgia, USA

^2^EMTEC IT, ExxonMobil Technical Computing Company, Annandale, New Jersey, USA

^3^Department of Chemistry, University of Georgia, Athens, Georgia, USA

^4^Department of Chemistry and Biochemistry, Auburn University, Auburn, Alabama, USA

^5^Energy Sciences, ExxonMobil Technology & Engineering Company, Annandale, New Jersey, USA

^6^Biomedical Sciences, ExxonMobil Technology & Engineering Company, Annandale, New Jersey, USA

^*^email: [whitman@uga.edu](mailto:whitman@uga.edu); [yuchen.liu@exxonmobil.com](mailto:yuchen.liu@exxonmobil.com)

**Supplementary Tables**

**Supplementary Table 1. Analysis of major posttranslational modifications (PTMs) in McrA of the purified recombinant MCR_aeo_ and MCR_mar_ by LC-MS/MS.** ^a^The Flag-Strep_2_ tag was added to the C-terminus of McrA (CA), the N-terminus of McrB (NB), or the N-terminus of McrG (NG) of the full *mcrBDCGA* operon. ^b^The Flag-Strep_2_ tag was added to the N-terminus of McrG position of the incomplete *mcr* operons. ^c^The site of the Me-Gln modification was identified on the pepsin digested peptides. ^d^The mass of long tryptic peptides were indicative of methylation, but the site of methylation was not identified.

| **MCR_aeo_** | **TGly447** | **Me-His260** | **Me-Arg274** | **Me-Gln402** |
| --- | --- | --- | --- | --- |
| NG-tagged^a^ | + | + | + | +^c^ |
| CA-tagged^a^ | + | + | + | +^d^ |
| NB-tagged^a^ | + | + | + | +^d^ |
| *mcrBDGA*^b^ | + | + | + | +^d^ |
| *mcrBCGA*^b^ | + | + | + | +^d^ |
| *mcrBGA*^b^ | + | + | + | +^d^ |
| **MCR_mar_** | **TGly448** | **Me-His261** | **Me-Arg275** | **Me-Gln403** |
| NG-tagged^a^ | + | + | + | +^c^ |

**Supplementary Table 2. Intact protein mass spectrometry of recombinant MCR_aeo._**

| **Complex I** | | | **Complex II** | | |
| --- | --- | --- | --- | --- | --- |
| **m/z** | **z** | **M (Da)** | **m/z** | **z** | **M (Da)** |
| 9020.614 | 32 | 288627.7 | 9154.509 | 31 | 283758.8 |
| 9304.076 | 31 | 288395.3 | 9458.609 | 30 | 283728.3 |
| 9612.052 | 30 | 288331.6 | 9784.458 | 29 | 283720.3 |
| 9948.828 | 29 | 288487 | 10136.17 | 28 | 283784.8 |
| 10300.14 | 28 | 288375.9 |  |  |  |
|  | Avg | 288443.5 |  | Avg | 283748.0 |
|  | Std Dev | 117.5 |  | Std Dev | 29.6 |

**Supplementary Table 3. General information on the source of *mcr* genes studied by heterologous expression.** ^a^The genome accession numbers are from GenBank assembly or RefSeq assembly.

| **Genome** | **Accession No.^a^** | ***mcr* operon** | **Origin** | **Ref.** |
| --- | --- | --- | --- | --- |
| ANME-1_BS | FP565147 | *BGA* | Black Sea microbial mat, Crimean area | ^1^ |
| ANME-1_G37 | GCA_003194425.1 | *BGA* | Enrichment culture (37 ^o^C) from Guaymas Basin hydrothermal sediment, Mexico | ^2^ |
| ANME-2b_HR1 | GCA_002926195.1 | *BDCGA* | Hydrate Ridge methane seep sediment, USA | ^3^ |
| ANME-2c_E20 | GCA_003194445.1 | *BDCGA* | Shallow-water seeps off the Island Elba, Italy | ^2^ |
| ANME-2c S7142MS2 | GCA_003336385.2 | *BDCGA* | Santa Monica Basin sediment, USA | ^3^ |
| ANME-2c WYZ-LMO14 | GCA_003601535.1 | *BDCGA* | Guaymas Basin hydrothermal sediment, Mexico | ^4^ |
| *Ca*. Ethanoperedens thermophilum E50 | GCA_905171685.1 | *BGA* | Enrichment culture (50 ^o^C) from Guaymas Basin hydrothermal sediment, Mexico | ^5^ |

**Supplementary Figures**

**
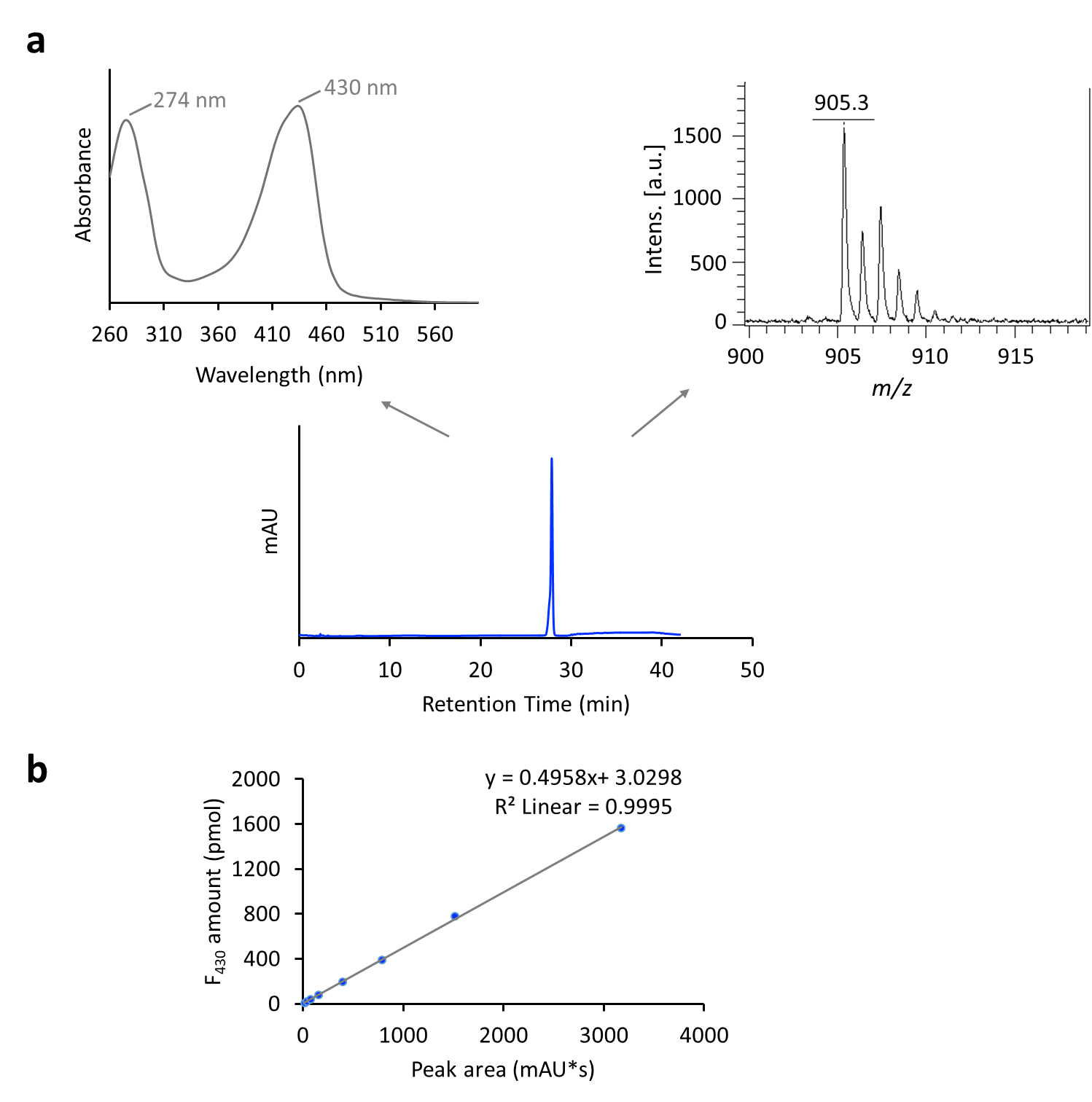
**

**Fig. S1 Purification and characterization of coenzyme F_430_ from recombinant MCR. a**, Reversed-phase HPLC chromatography of methanol-extracted coenzyme F_430_ analyzed by UV-visible spectroscopy and MALDI-TOF-MS. **b**, The standard curve for coenzyme F_430_ quantification.

**
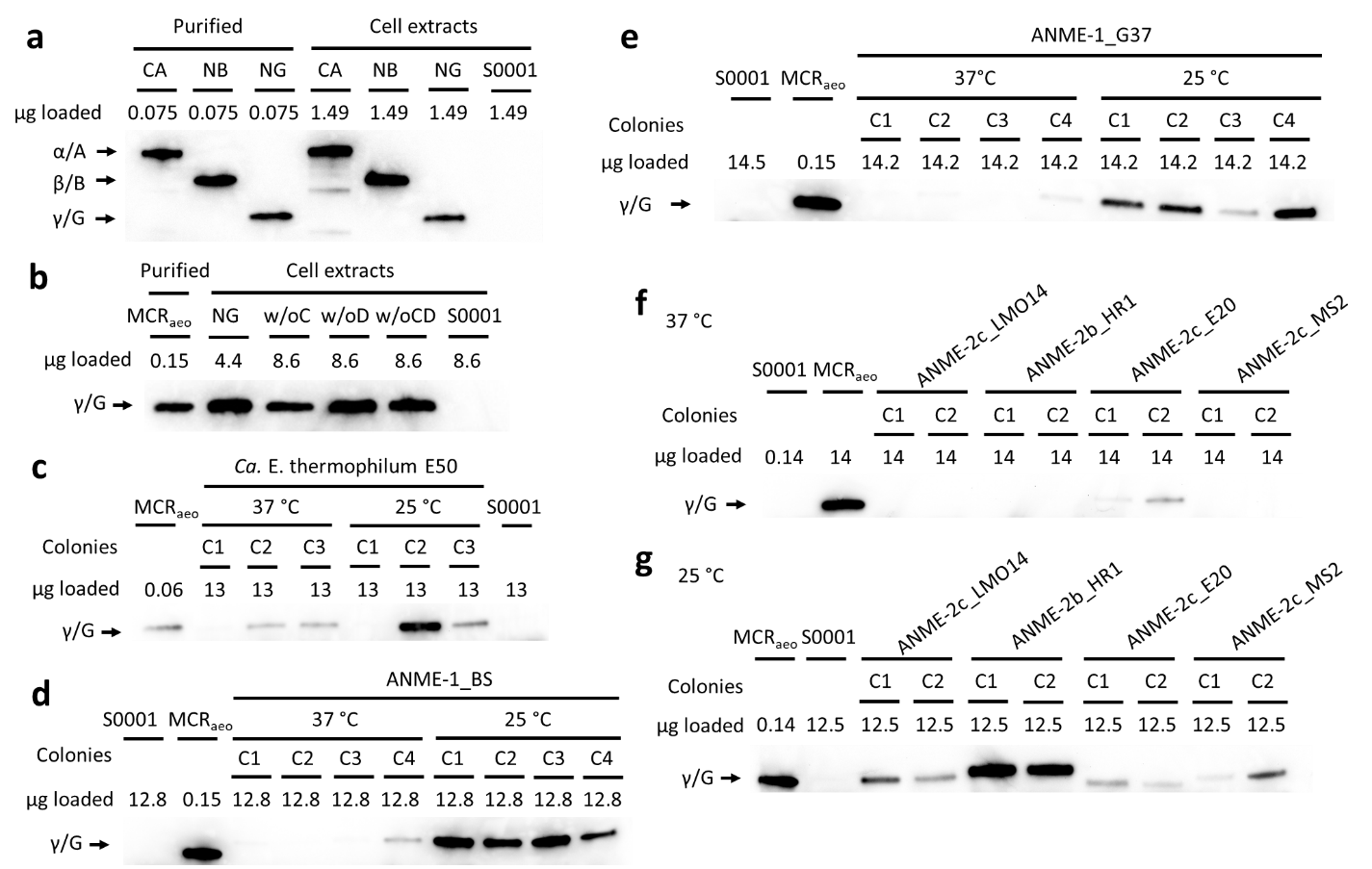
**

**Fig. S2 Expression of Flag-tagged MCRs in *M. maripaludis* from methanogens and uncultured archaea**. The amount of protein loaded on each gel is indicated. S0001 lacked the Flag-Strep_2_-tagged recombinant MCR and served as the negative control. MCR_aeo_, purified recombinant Flag-tagged *M. aeolicus* rMCR was used as a positive control. **a**, The Flag tag was added to the C-terminal of McrA (CA), N-terminal of McrB (NB), and N-terminal of McrG (NG) under the control of the P*pst* promoter. **b**, Relative expression of the truncated operons. NG, rMCR_aeo_ with complete *mcrBDCGA* operon; w/oC, rMCR_aeo_ with incomplete *mcrBDGA* operon; w/oD, rMCR_aeo_ with incomplete *mcrBCGA* operon; w/oCD, rMCR_aeo_ with incomplete *mcrBGA* operon. **c**, Expression of recombinant *Ca.* E. thermophilum E50 ECR in *M. maripaludis*. Western blots with anti-Flag antisera to cell free extracts from clones containing the expression plasmid for ANKA_E50 MCR with the N-terminal of G tagged following growth at 37 °C and 25 °C. Three colonies [C1-C3] were screened. **d**, Expression of recombinant ANME-1_BS MCR in *M. maripaludis*. Western blots with anti-Flag antisera to cell free extracts from clones containing the expression plasmid for ANME-1_BS MCR with the N-terminal of G tagged following growth at 37 °C and 25 °C. Multiple colonies [C1-C4] were screened. **e**, Expression of recombinant ANME-1_G37 MCRs in *M. maripaludis*. Western blots with anti-Flag antisera to cell free extracts from clones containing the expression plasmids for ANME-1_BS (d) and ANME-1_G37 (e), all tagged on the G subunit following growth at 37 °C and 25 °C. Multiple colonies [C1-C4] of each were screened. **f**,**g**, Expression of recombinant ANME-2 MCRs in *M. maripaludis*. Western blots with anti-Flag antisera to cell free extracts from clones containing the expression plasmids for four ANME-2 MCRs, ANME-2b_HR1, ANME-2c_LMO14, ANME-2c_E20 and ANME-2c_MS2, all tagged on the G subunit following growth at 37 °C and 25 °C. Two colonies [C1-C2] of each were screened.

**Fig. S3. The *mcr* expression levels of the *M. maripaludis* strain recombinantly expressing the ANME-1_G37 *mcrBGA* operon during growth at 37 ^o^C.** The *mcr* mRNA levels were measured by qRT-PCR and depicted by copy numbers per 30 ng of the purified total RNA. The error bars represent standard deviations from three PCR reactions.

**
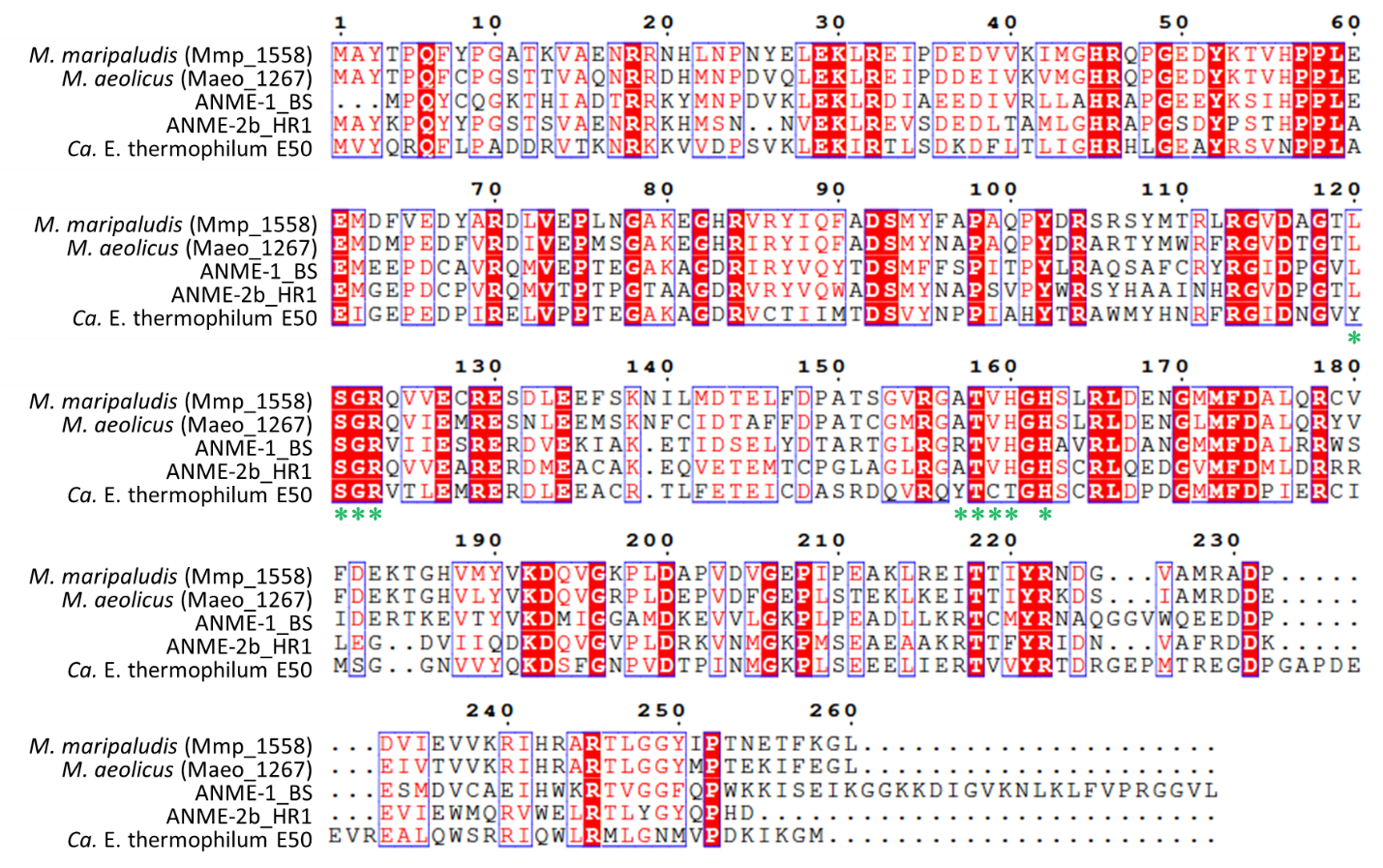
**

**Fig. S4 Sequence alignment of McrG homologs.** McrG homologs from five genomes including *M. maripaludis*, *M. aeolicus*, ANME-1_BS, ANME-2b_HR1, and *Ca.* Ethanoperedens thermophilum E50. The F_430_ binding sites (residues 120-123, 157-160, and 162 of Mmp_1558) are highlighted by * (in green) under the sequence.

**
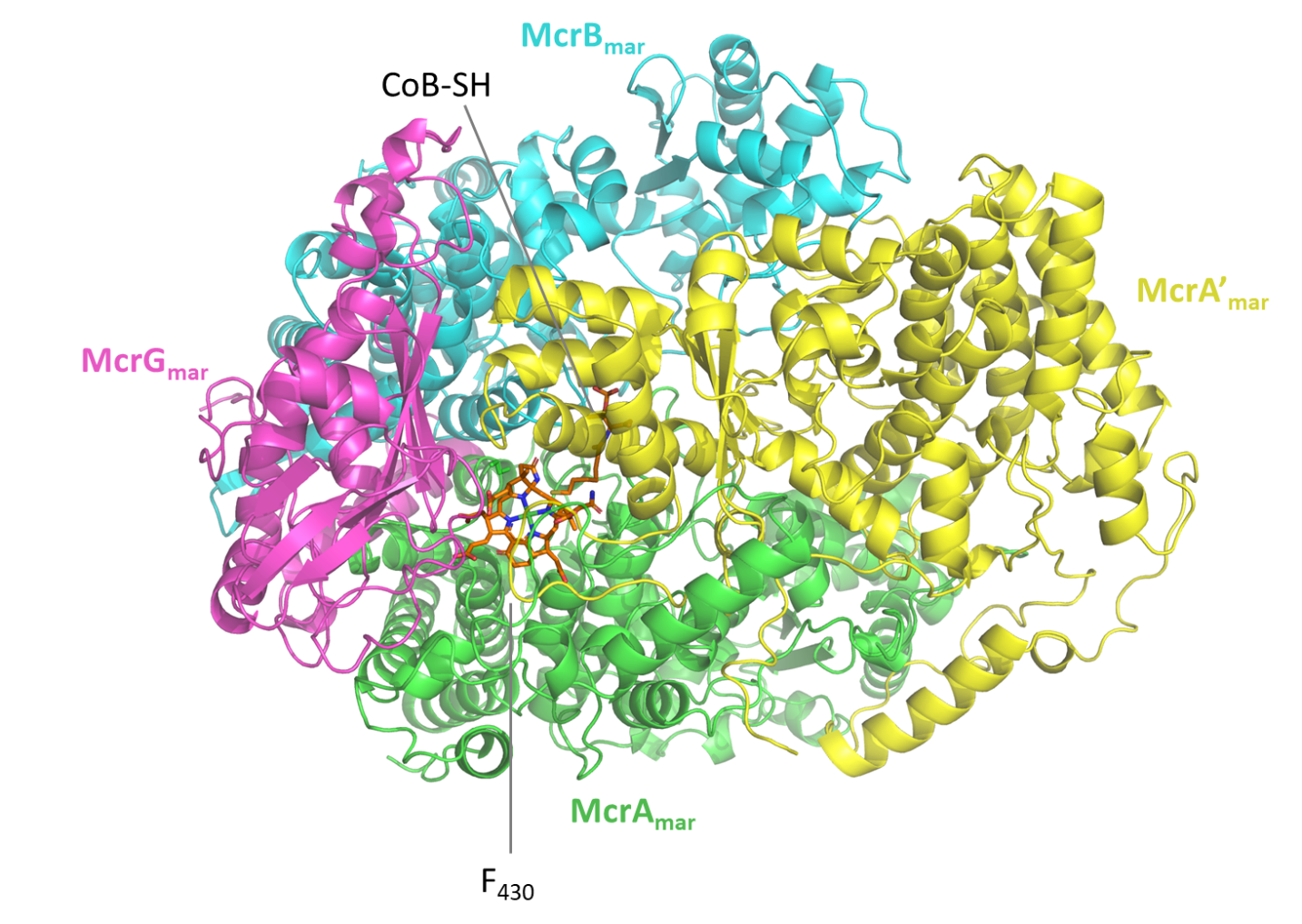
**

**Fig. S5 The best scored *Methanococcus maripaludis* MCR model out of 40,000 models generated with RosettaCM.** The protein subunits are presented in the cartoon, and F_430_ and CoB-SH are depicted in stick models. Only one active site composed of the *M. maripaludis* McrA (green), A’ (yellow), B (cyan), G (magenta), and one F_430_ are shown for clarity.

**Fig. S6 Uncropped western blots and gels**

Related to Figs. 2, 3, 4, 5 & 6 and Supplementary Fig. S2.

Figure 2b


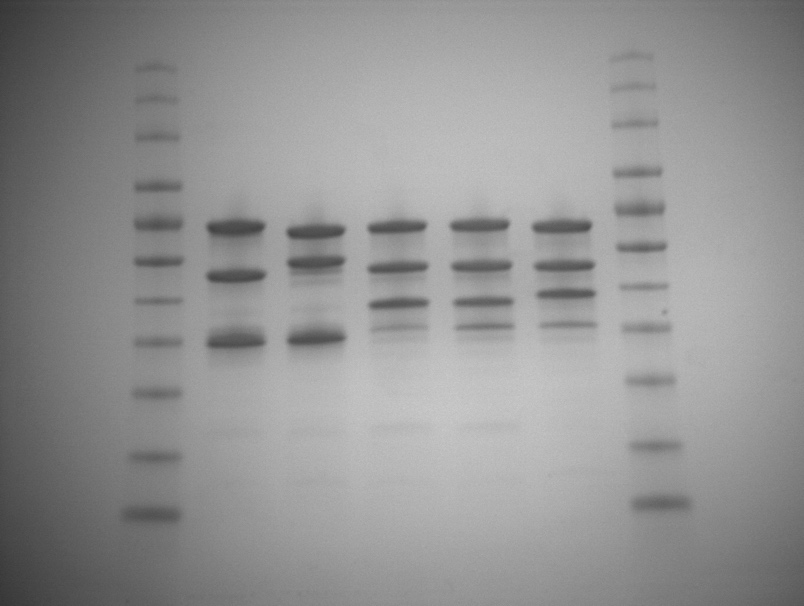


kDa

250

180

130

95

72

55

43

34

26

17

10

NG-tag

MCR_mar_

NG-tag

MCR_aeo_

NG-tag

MCR_aeo_

CA-tag

MCR_aeo_

NB-tag

MCR_aeo_

Figure 3a Figure 3b


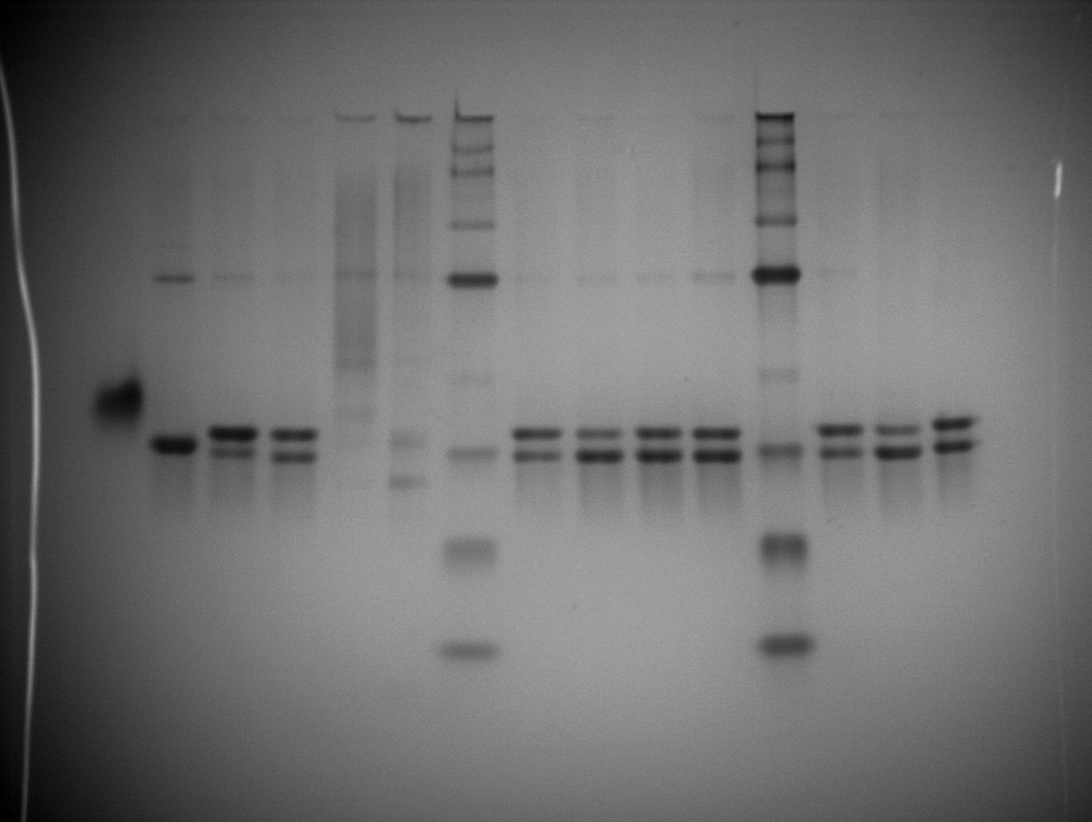
 **
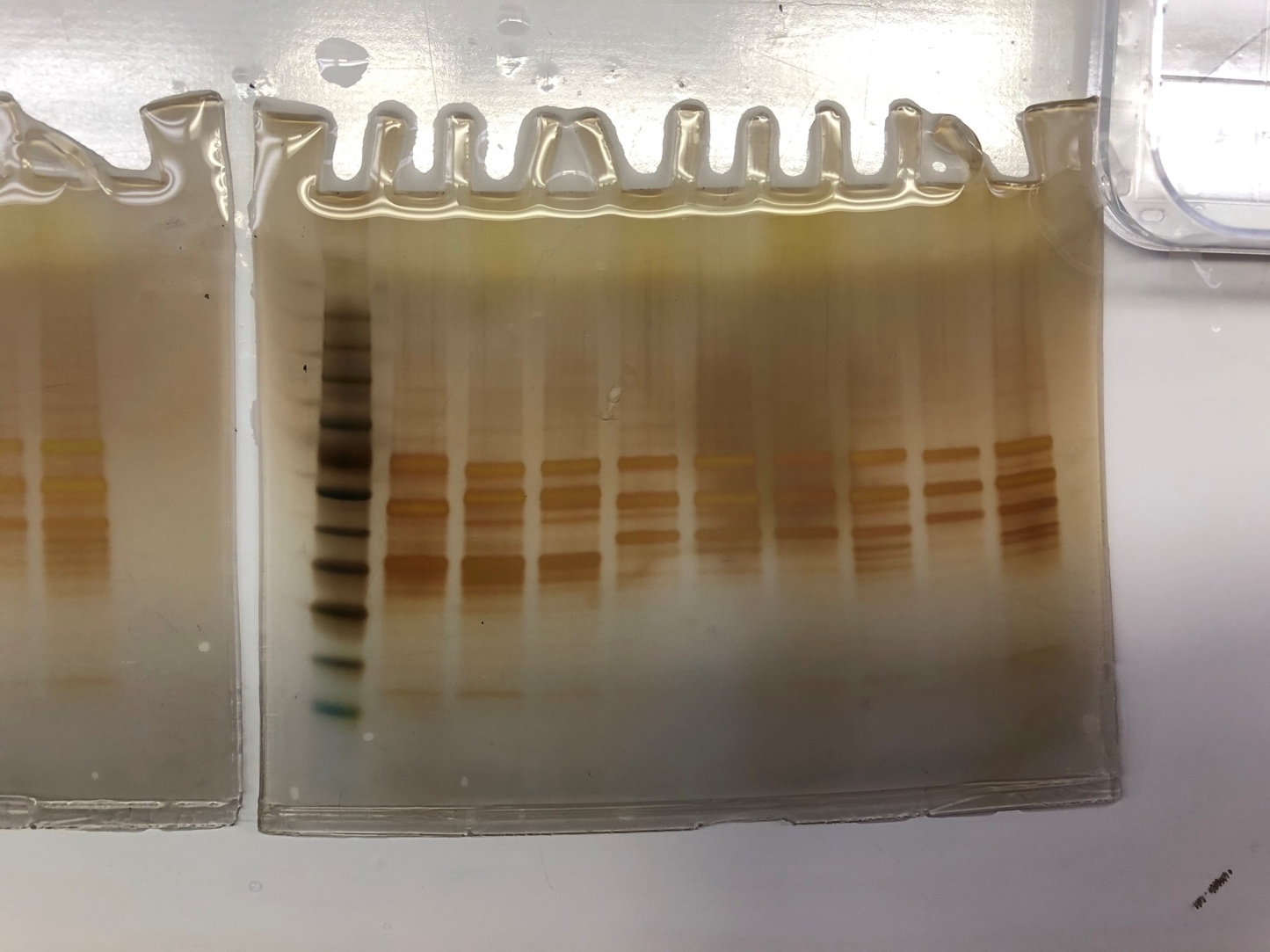
**

I II

MCR_aeo_ MCR_mar_

kDa

1236

1048

720

480

242

146

66

20

kDa

250

180

130

95

72

55

43

34

26

17

10

Figure 4a


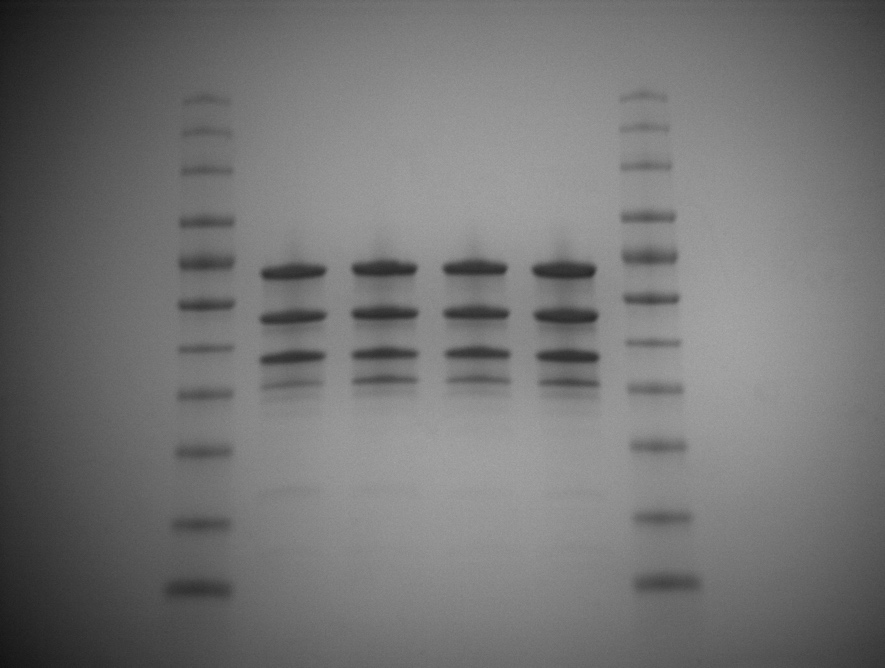


*mcrBGA*

*mcrBCGA*

*mcrBDGA*

*mcrBDCGA*

kDa

250

180

130

95

72

55

43

34

26

17

10

Figure 5a-McrC_aeo_ and 5b-McrD_aeo_ Figure 5a-McrC_mar_

kDa

250

150

100

75

50

37

25

20

15


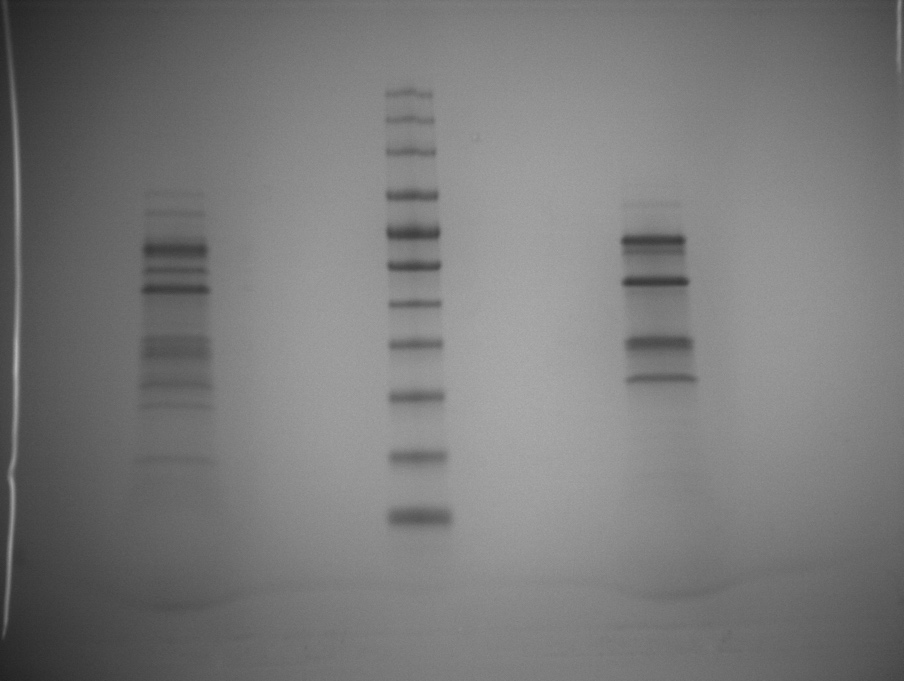

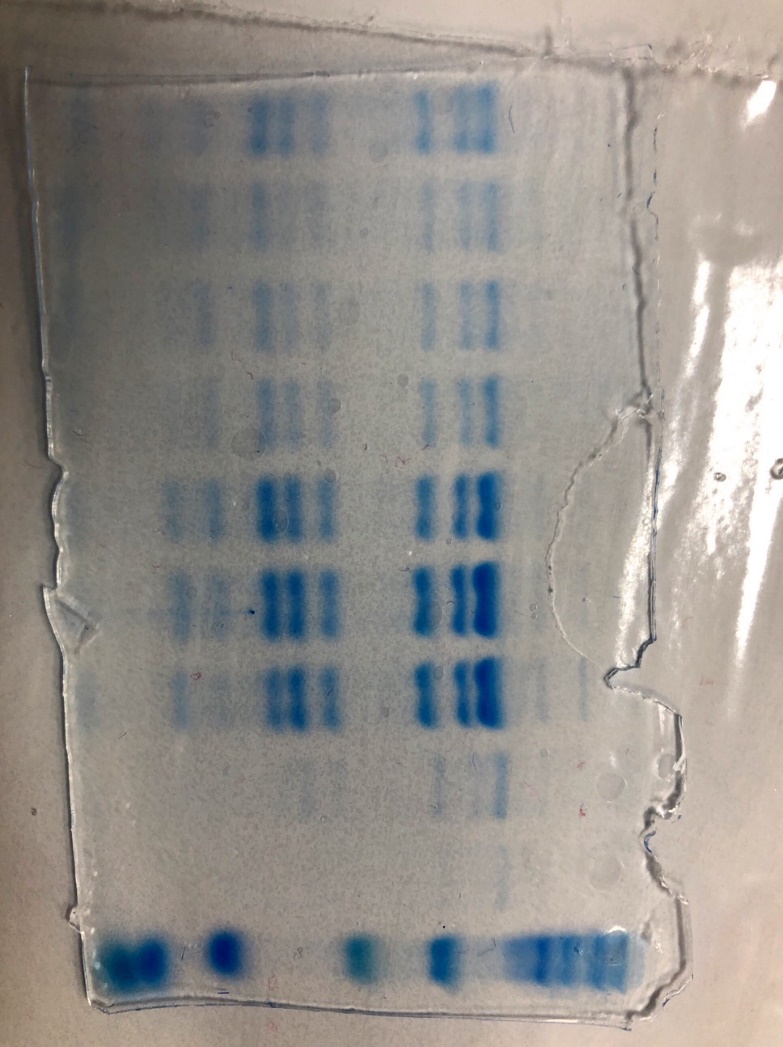


McrC_mar_

McrD_aeo_

McrC_aeo_

kDa

250

180

130

95

72

55

43

34

26

17

10

Figure 6b Figure 6c Figure 6d


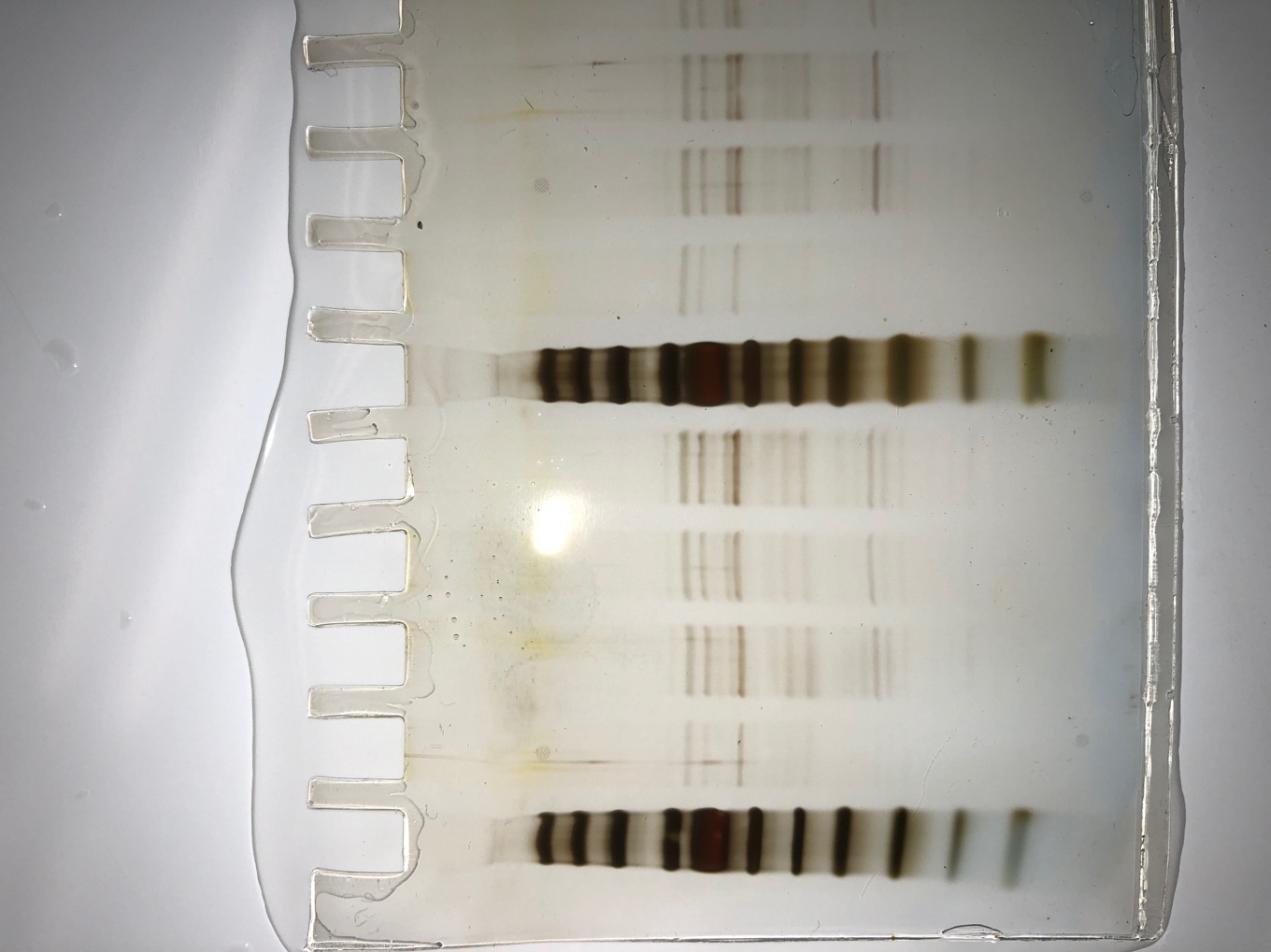

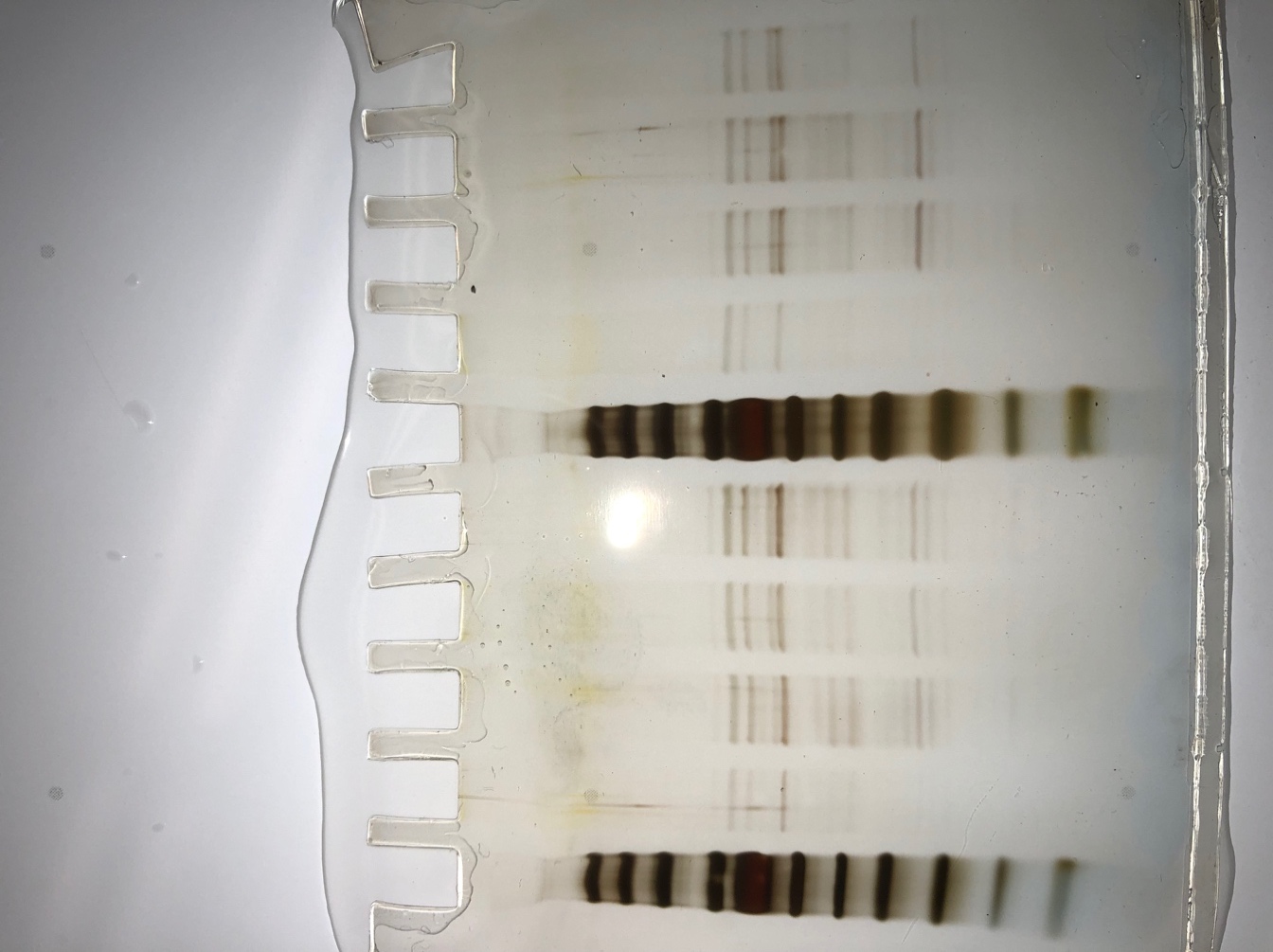

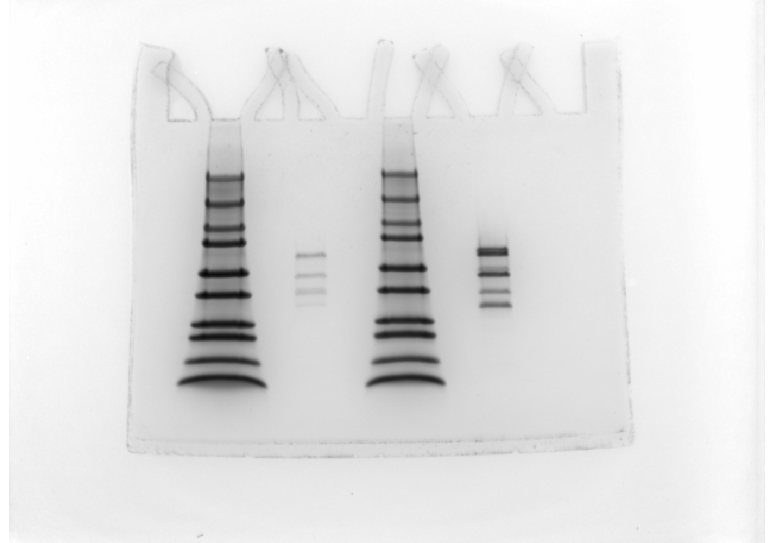


MCR_ANME-2_HR1_

ECR_E50_

MCR_ANME-1_BS_

kDa

250

180

130

95

72

55

43

34

26

17

10

kDa

250

180

130

95

72

55

43

34

26

17

10

kDa

250

180

130

95

72

55

43

34

26

17

10

Figure 4d. Western blot with anti-Flag antisera to cell free extracts of clones containing the plasmids for four MCRs, NG, w/oC, w/oD, w/oCD. NG, MCR_aeo_ with complete *mcrBDCGA* operon; w/oC, MCR_aeo_ with incomplete *mcrBDGA* operon; w/oD, MCR_aeo_ with incomplete *mcrBCGA* operon; w/oCD, MCR_aeo_ with incomplete *mcrBGA* operon. The amount of protein loaded on the gel is indicated. S0001 lacked the Flag-Strep_2_-tagged recombinant MCR and served as the negative control. MCR_aeo_, purified recombinant Flag-tagged *M. aeolicus* MCR was used as a positive control. Multiple colonies [C1-C3] were screened.


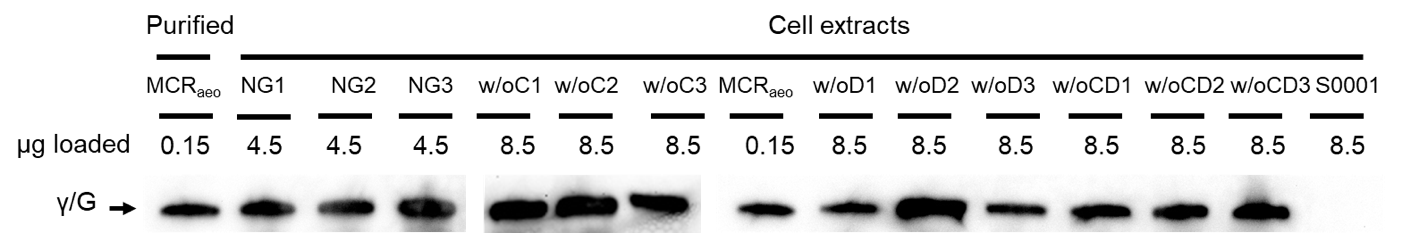


MCRaeo, *mcrBDCGA* (NG1-3)


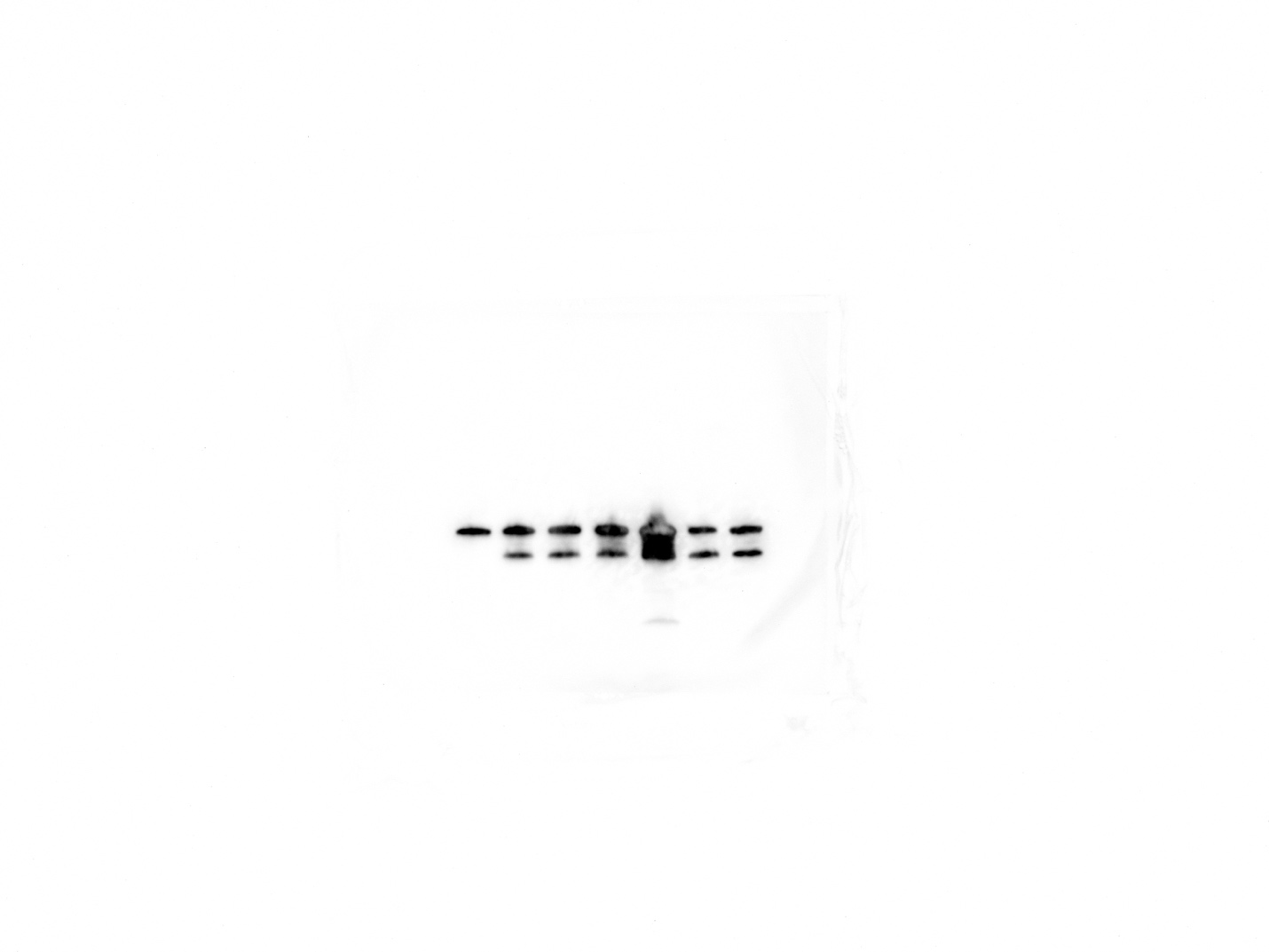


30 kDa

γ/G

non-specific band

MCRaeo, *mcrBDGA* (w/oC 1-3)


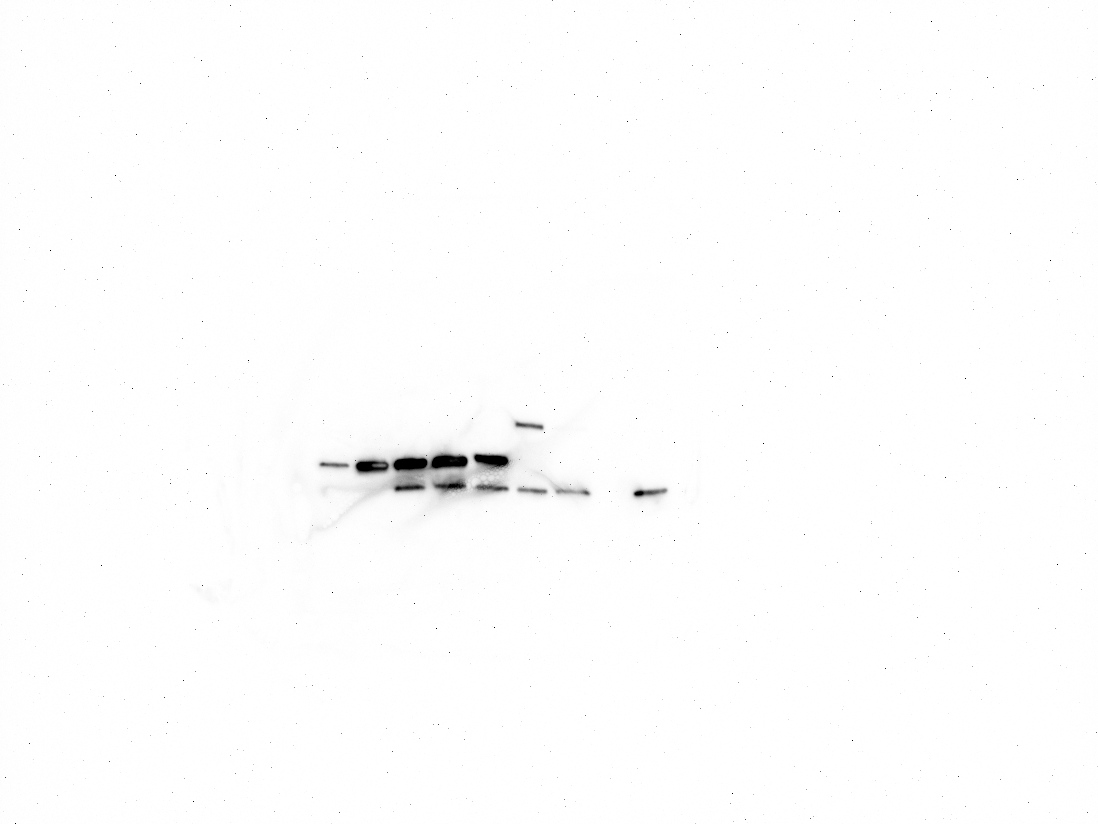


γ/G

non-specific band

30 kDa

MCRaeo, *mcrBCGA* (w/oD 1-3), *mcrBGA* (w/oCD1-3)


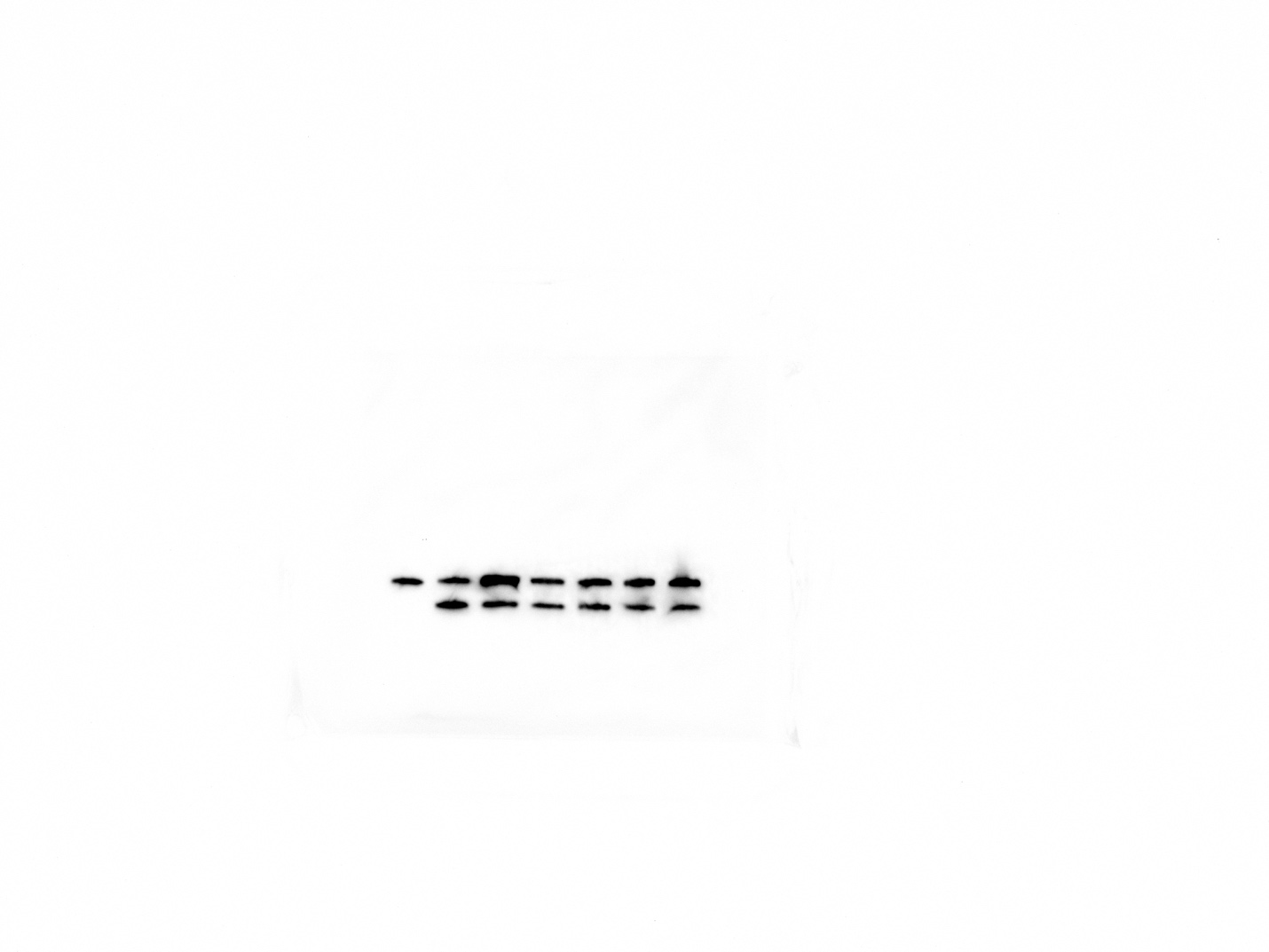


γ/G

non-specific band

30 kDa

Figure S2a


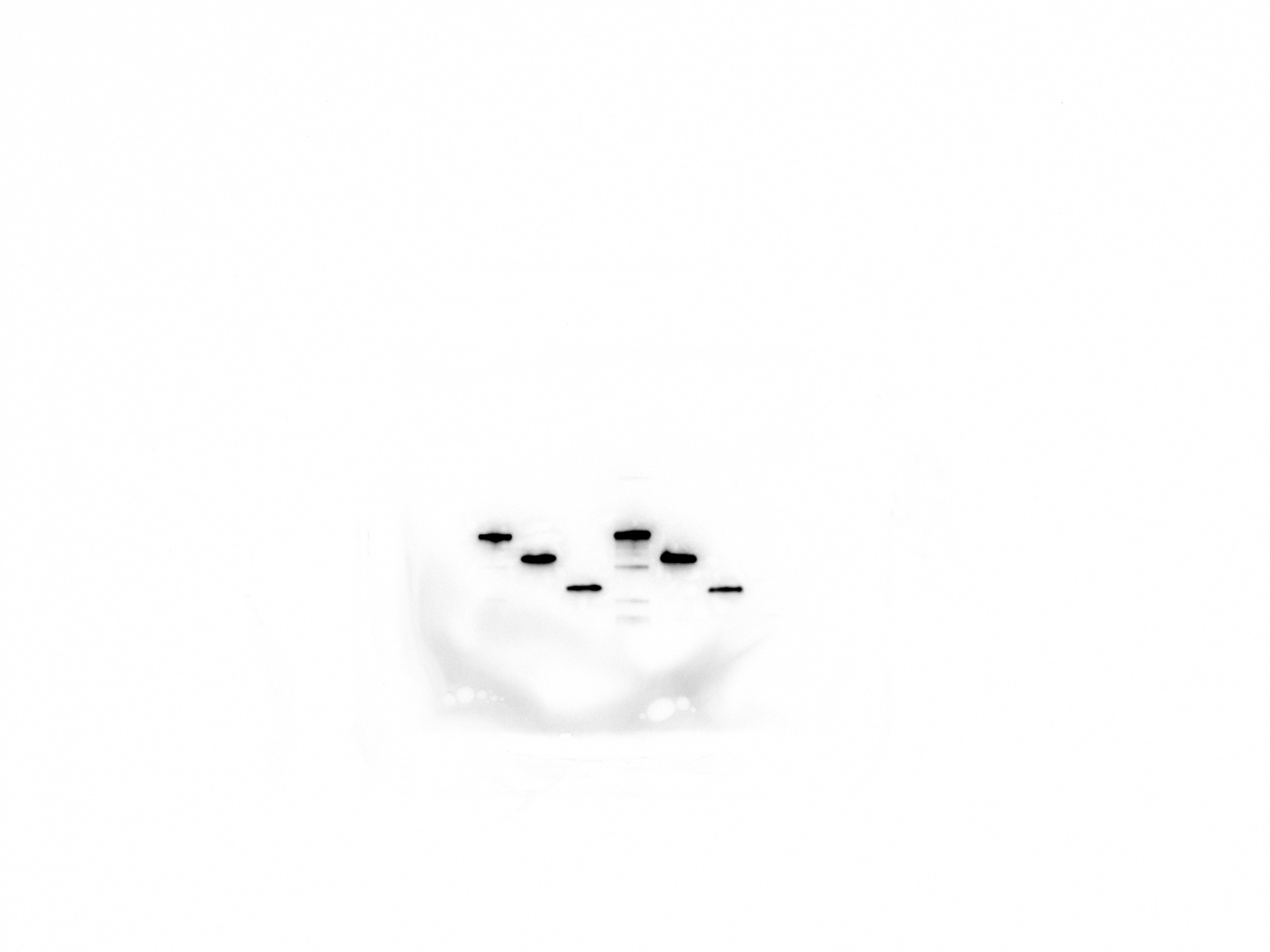


60 kDa

50 kDa

30 kDa

α/A

β/B

γ/G

Figure S2b


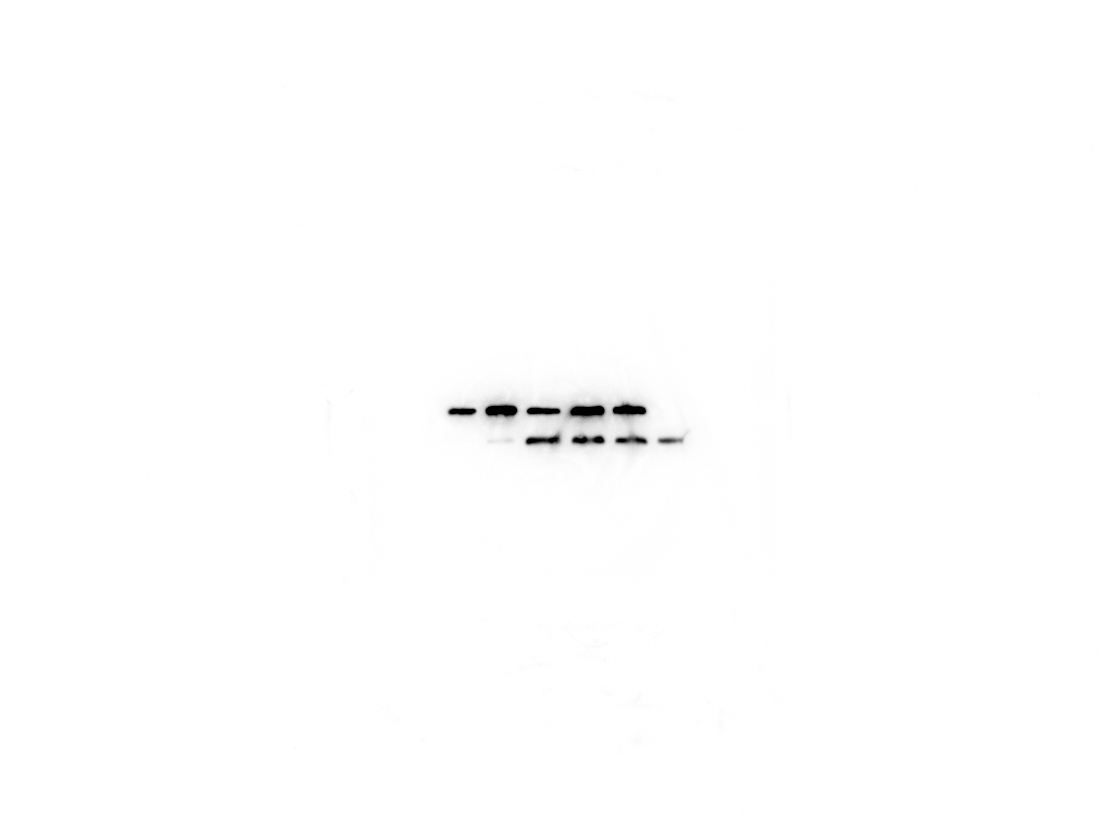


30 kDa

γ/G

non-specific band

Figure S2c


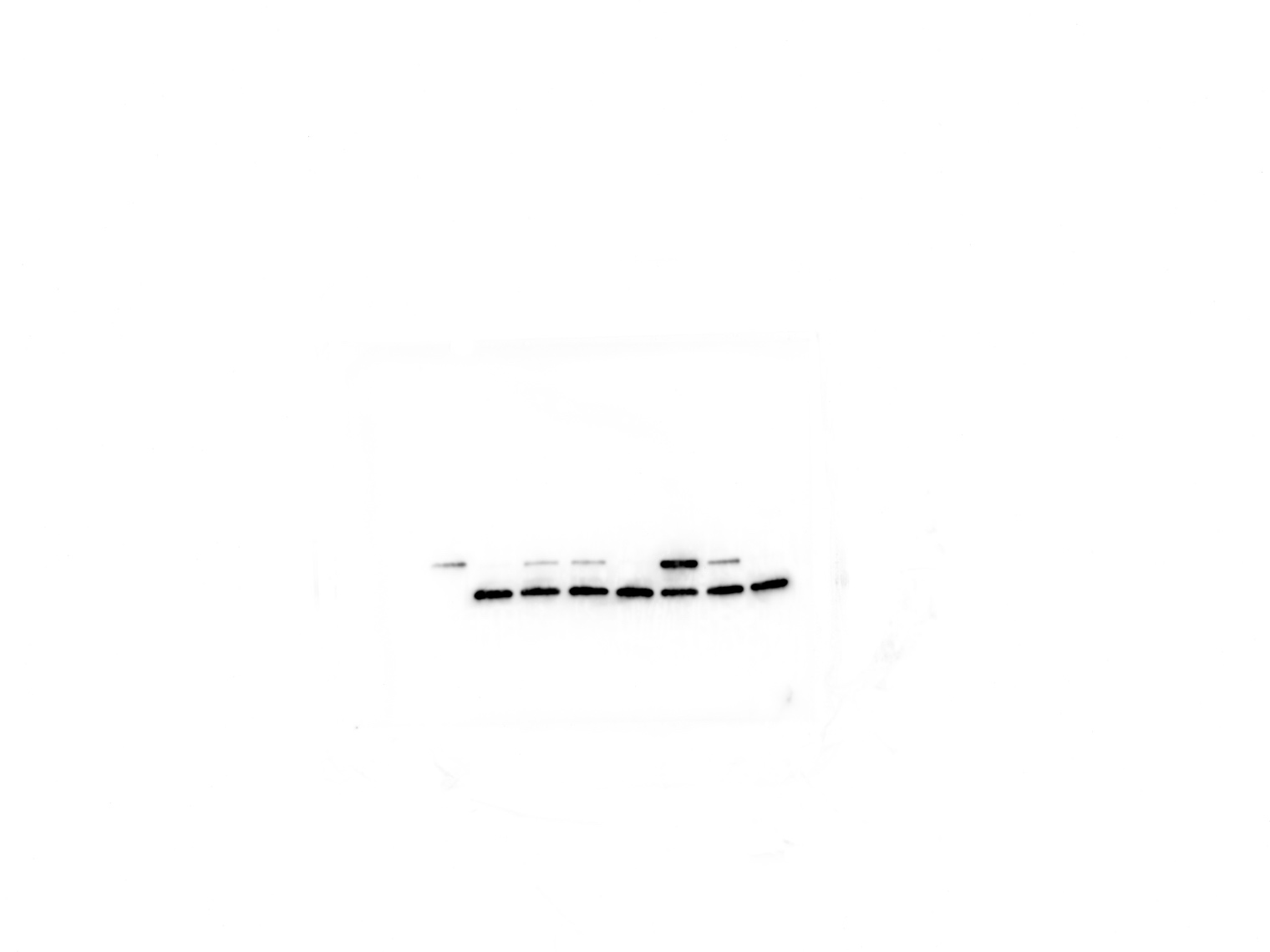


γ/G

non-specific band

30 kDa

Figure S2d


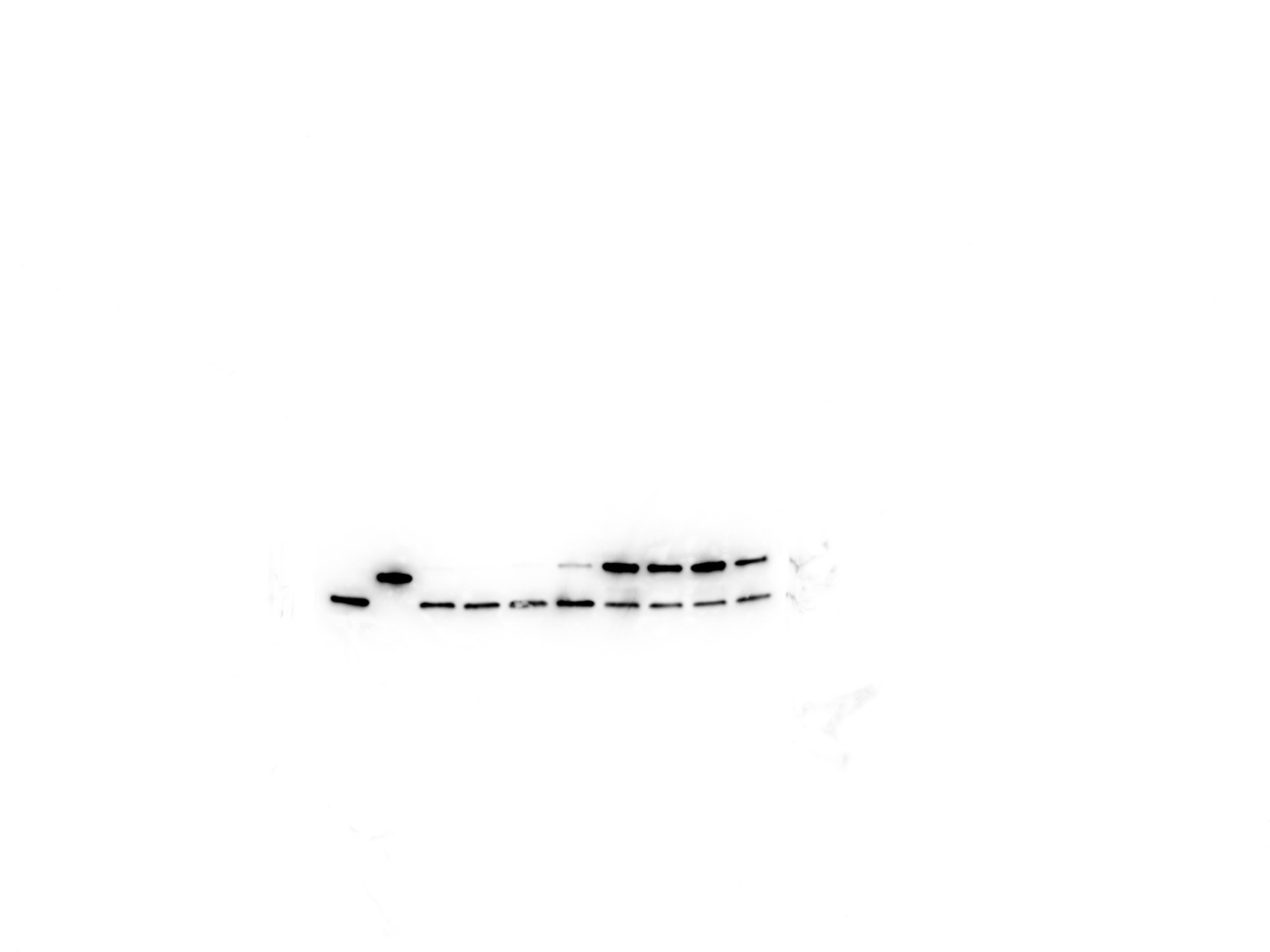


30 kDa

γ/G

non-specific band

Figure S2e


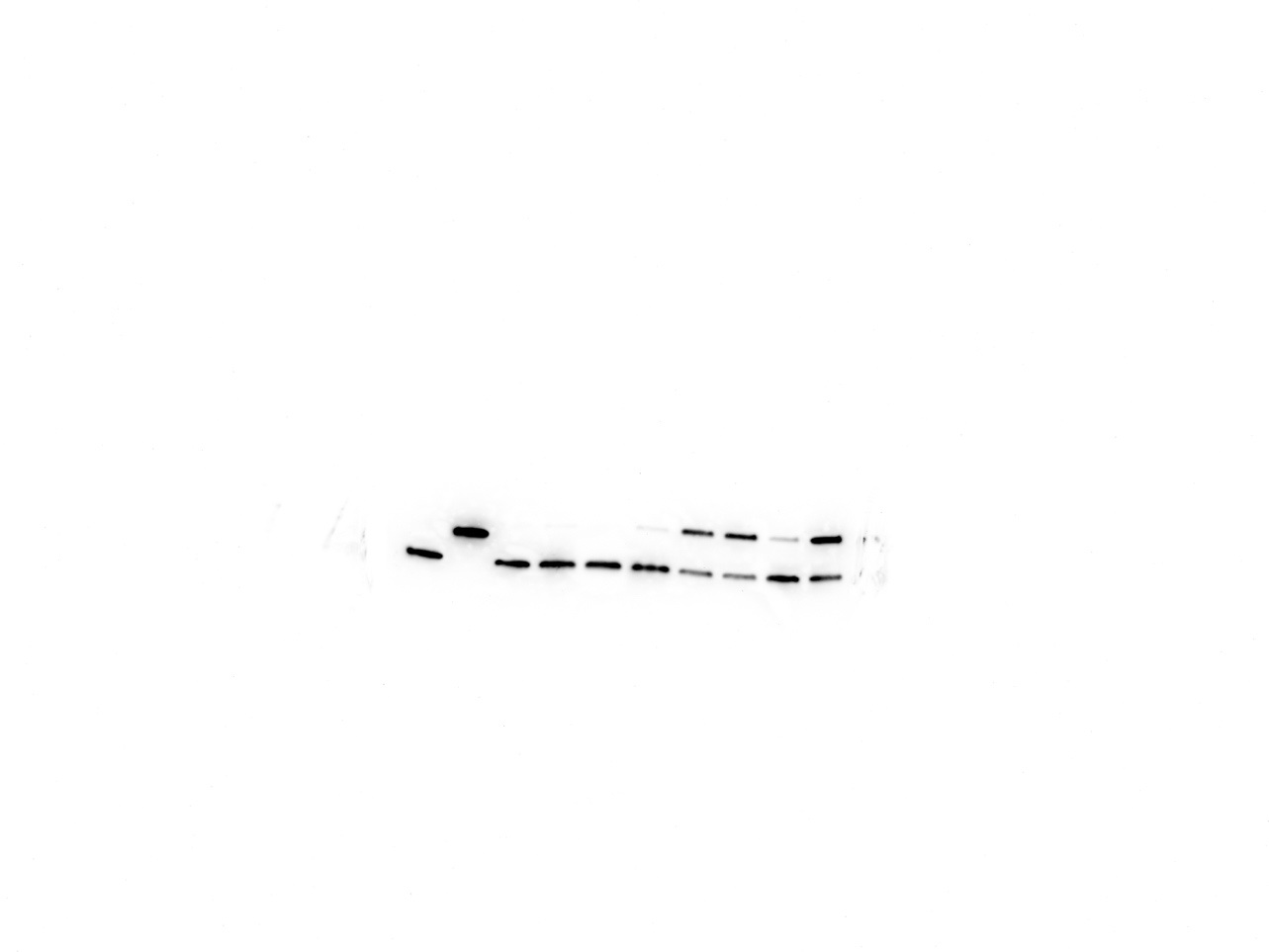


γ/G

non-specific band

30 kDa

Figure S2f


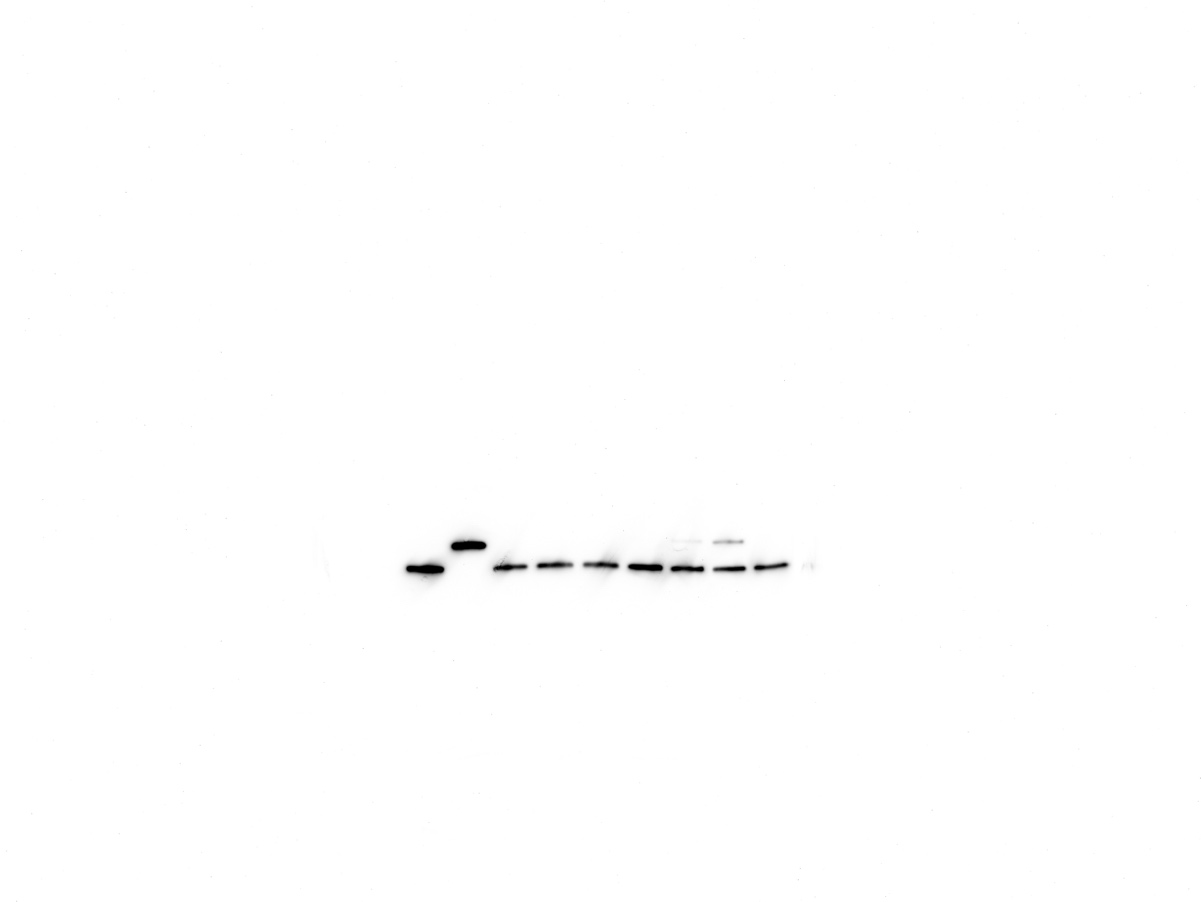


γ/G

non-specific band

30 kDa

Figure S2g


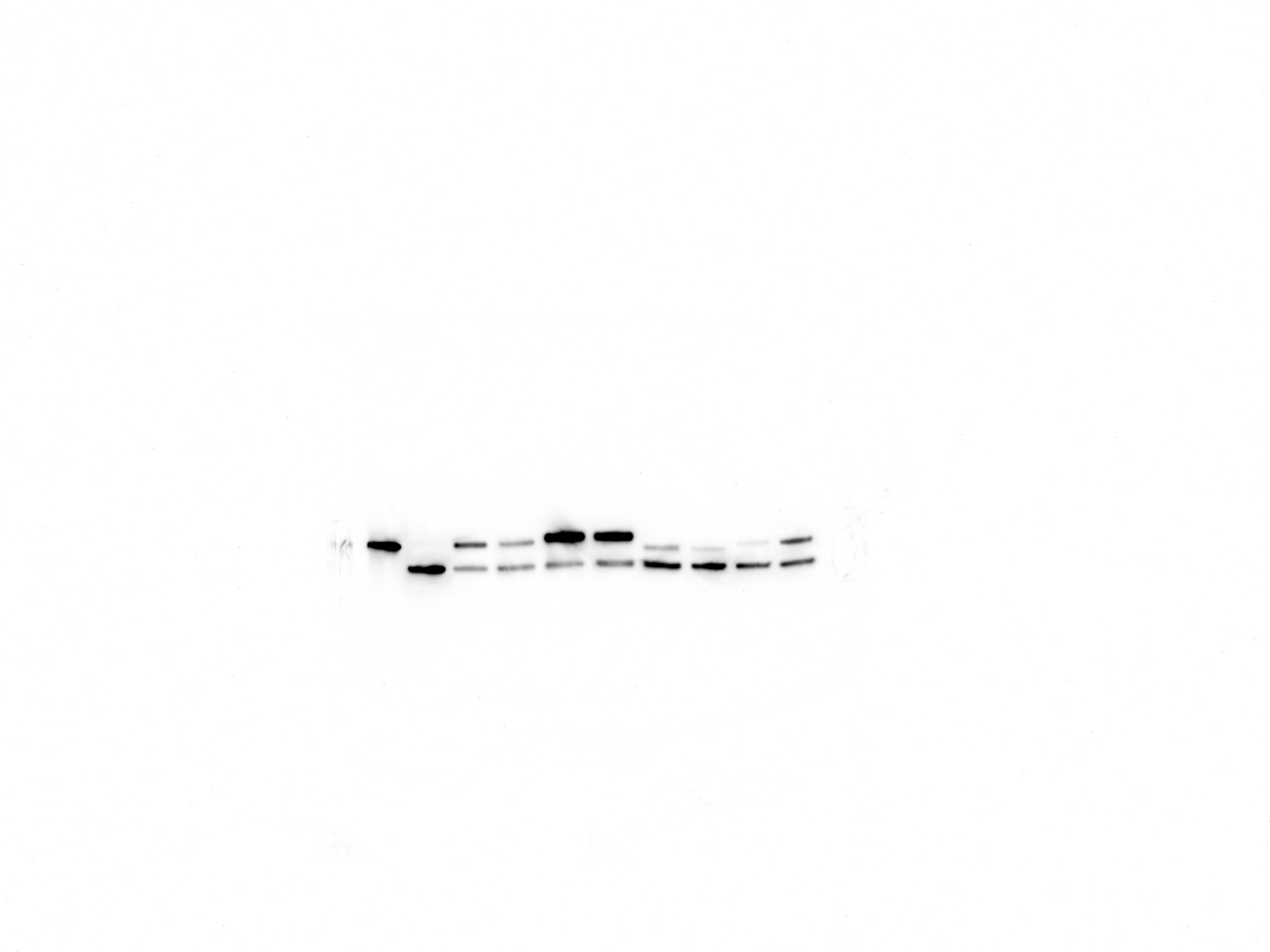


γ/G

non-specific band

30 kDa

**Supplementary references**

1. Meyerdierks, A. et al. Metagenome and mRNA expression analyses of anaerobic methanotrophic archaea of the ANME-1 group. *Environ Microbiol* **12**, 422-439 (2010).

2. Krukenberg, V. et al. Gene expression and ultrastructure of meso- and thermophilic methanotrophic consortia. *Environ Microbiol* **20**, 1651-1666 (2018).

3. Yu, H. et al. Comparative genomics and proteomic analysis of assimilatory sulfate reduction pathways in anaerobic methanotrophic archaea. *Front Microbiol* **9**, 2917 (2018).

4. Wang, Y., Feng, X., Natarajan, V.P., Xiao, X. & Wang, F. Diverse anaerobic methane- and multi-carbon alkane-metabolizing archaea coexist and show activity in Guaymas Basin hydrothermal sediment. *Environ Microbiol* **21**, 1344-1355 (2019).

5. Hahn, C.J. et al. “Candidatus Ethanoperedens,” a Thermophilic Genus of Archaea Mediating the Anaerobic Oxidation of Ethane. *mBio* **11**, e00600-00620 (2020).
