# Supplementary material for: Expression of divergent methyl/alkyl coenzyme M reductases from uncultured archaea
Source: Commun Biol. 2022 Oct 20;5:1113. doi: 10.1038/s42003-022-04057-6 (PMC9584954; doi:10.1038/s42003-022-04057-6)
Supplement: Supplementary file 3 — Description of Additional Supplementary Files [file 42003_2022_4057_MOESM3_ESM.docx]

**Description of Additional Supplementary Files**

**File name:** Supplementary Data 1

**Description:** The *mcr* operons in archaea

**File name:** Supplementary Data 2

**Description:** Raw mass spectrometry data for quantification of the relative abundance

**File name:** Supplementary Data 3

**Description:** Raw data for figure plotting
